# Perceived Social Change, Parental Control, and Family Relations: A Comparison of Chinese Families in Hong Kong, Mainland China, and the United States

**DOI:** 10.3389/fpsyg.2017.01671

**Published:** 2017-10-09

**Authors:** Joey Fung, Joanna J. Kim, Joel Jin, Qiaobing Wu, Chao Fang, Anna S. Lau

**Affiliations:** ^1^School of Psychology, Fuller Theological Seminary, Pasadena, CA, United States; ^2^Department of Psychology, University of California, Los Angeles, Los Angeles, CA, United States; ^3^Department of Applied Social Sciences, The Hong Kong Polytechnic University, Hong Kong, Hong Kong; ^4^School of Psychology, Beijing Normal University, Beijing, China

**Keywords:** social change, autonomy support, psychological control, child acceptance, family conflict

## Abstract

This study examined the relationship between perceived social change, parental control and family relations in a sample of 419 4th and 5th grade children and their mothers who are of Chinese descent but reside in three different contexts: Los Angeles (LA), Hong Kong (HK), and Beijing (BJ). HK mothers endorsed the highest levels of psychological control and the lowest levels of autonomy support compared to BJ and LA mothers. Perceived social change as measured by mothers’ endorsement of new values and ideologies was associated with increased use of both autonomy support and psychological control. Results of the mediation analyses suggested that perceived social change explained differences between LA and HK mothers in autonomy support, but group differences in psychological control were magnified when perceived social change was accounted for. Finally, whereas autonomy support was associated with higher levels of child perceived acceptance in HK and LA, psychological control was associated with greater family conflict in BJ and LA. Findings suggested that as families undergo urbanization or social change, it may shift the implications of traditional strategies that are intended to socialize the child toward interpersonal attunement. Overall, the study highlights the importance of moving beyond ethnic-group or cross-national comparisons to investigate the role of changing social and economic contexts in understanding differences in the use of parental control and their associations with family relations.

## Introduction

In Western theory and research on parenting, psychological control is often used to refer to parental intrusiveness, domination, or coercive control, with the inverse being parental support of autonomy (Pomerantz and Wang, 2009). In individualistic American culture, exerting psychological control, through inducing anxiety or guilt, shaming or withdrawing love to modify child behavior, is perceived as an intrusion upon the child’s sense of self and individuation, as it undermines the child’s attainment of self-reliance, self-expression, and emotional autonomy (Barber, 1996). On the other hand, supporting autonomy, by allowing the child to make independent choices or encouraging exchange of opinions, serves to foster the child’s individuality, self-efficacy, and self-determination. Indeed, cultural ideals in Western parenting tend to nurture children’s appropriate self-expression and self-esteem (Chao, 1995; Rothbaum et al., 2000). Consistent with this ethos, psychological control is often linked to lower wellbeing in children, such as depression, anxiety, and externalizing problems in Western samples (Barber et al., 1994; Pettit et al., 2001), whereas autonomy support is linked to enhanced emotional functioning, social skills, and academic competence (e.g., Gray and Steinberg, 1999; Silk et al., 2003).

Yet, there has been a longstanding discussion among cultural scholars as to whether the effects of parental control strategies may vary according to the cultural context in which they take place (e.g., Chao, 1994). In contrast to Western cultural contexts where autonomy and assertiveness are emphasized as desired end goals of child development, East Asian cultures prioritize emotional restraint, self-control, and maintaining interpersonal harmony. The optimal endpoint of development within more collectivistic culture, such as Chinese culture, is not necessarily to achieve independence from others, but to achieve interdependence through the fulfillment of collective goals for the benefit of the social unit, starting with the family (Greenfield et al., 2003). To that end, in collectivistic Chinese culture, parental psychological control functions as one of the socialization avenues through which children acquire and internalize the requisite self-control to adhere to behavioral and social expectations in any and all social contexts (Markus and Kitayama, 1991). By stressing the relational consequences of actions and drawing the child’s attention to the effects of their misbehavior on the parent’s emotions, psychological control strategies may promote the child’s sensitivity to the judgments and feelings of others and encourage accommodation to norms (Fung and Lau, 2012).

Given that parenting behaviors are shaped by socialization goals and priorities, research has documented significant differences in the use of parental control between Chinese national, Chinese American, and European American parents. Cross-national studies suggest that Chinese parents rely on strategies of love withdrawal, shaming, and guilt induction more so than their European American counterparts (Leung et al., 1994; Wu et al., 2002; Ng et al., 2014). Children in Mainland China, compared to children in the United States, report that their parents make more decisions about their personal issues (e.g., Qin et al., 2009) and use more psychological control and less autonomy support strategies (e.g., Cheung and Pomerantz, 2011). Similarly, children in Hong Kong describe their parents as more authoritarian and less authoritative (Leung et al., 1998) and demonstrate greater reliance on the use of psychological control (Fung and Lau, 2012) compared to European American children. Chinese immigrant adolescents also report that their parents exert more psychological control and less autonomy support than European American adolescents (e.g., Chao and Aque, 2009). These differences between Chinese and European American parents emerge early in child development. Chinese parents in Hong Kong, Taiwan, and China report greater use of psychological and behavioral control of preschool aged children compared to European American parents (Wu et al., 2002). However, little work to date has been done to examine differences in the use of parental control between families of Chinese descent who reside in different societal context (e.g., Chinese families in Mainland China versus Chinese families in Hong Kong versus immigrant Chinese families in the United States). One of the goals of the present study is to directly compare Chinese parents reported use of parental control across the three societal contexts.

Beyond mean differences in the use of parental control, the literature also suggests differences in its associations with child adjustment and family climate across cultural contexts. While restrictive parental control is often associated with lower levels of perceived parental acceptance within European American families (Garber and Flynn, 2001), parental control has been linked to greater perceptions of parental warmth and acceptance, and lower levels of family conflict in Asian and Asian American families (Rohner and Pettengill, 1985; Kornadt, 1991; Nomura et al., 1995). In terms of psychological control and child emotion functioning, some studies have found that psychological control is associated with increased internalizing and externalizing problems in children from both mainland China and the United States (e.g., Barber et al., 2006; Wang et al., 2007) as well as Chinese immigrant adolescents (e.g., Chao and Aque, 2009). Other studies, however, have found that the associations between psychological control and child internalizing and externalizing problems to be non-significant in a Hong Kong sample (Fung and Lau, 2009) and in Mainland China when comparing across boys and girls (Olsen et al., 2002). In contrast, parental autonomy support has received near universal support as promoting child wellbeing across cultures. Autonomy support predicts children’s enhanced emotional functioning and achievement in China and the United States (Vansteenkiste et al., 2005; Wang et al., 2007; Lekes et al., 2010), and in the United Kingdom and Hong Kong (Taylor and Lonsdale, 2010). Less is known about how parental autonomy support impacts family processes such as conflict or perceived acceptance, although it is reasonable to expect positive associations with favorable climate and available findings suggest autonomy support is linked with parental warmth (e.g., Fulton and Turner, 2008).

Apart from the observed East–West differences in the use and correlates of parental control, differences may also be observed based on social context and economic class. There are important differences among families within the Chinese diaspora that can be attributed to population-level changes in social and economic societal contexts, as well as individual-level transitions in the priorities of families. Greenfield (2009) articulated her theory of how changing socio-demographic ecologies alter cultural values and the resultant socialization and development of children. Globally, developing societies show increased movement from rural residence, informal education at home, subsistence economy, and low-technology environments to urban residence, formal schooling, commerce, and high-technology environments. Socioeconomic development has been associated with a shift from tight family relations to increased positive attitudes around children’s independence and autonomy outside the home (Kagitcibasi and Ataca, 2005). Social changes and economic developments that take place at the societal or community level may impact the unique experiences of individuals and families. In particular, certain traditional Chinese beliefs and norms may be deemed incompatible with the skill sets required that of the market-oriented society, increasingly emphasizing traditionally Western values and ideologies. In order for individuals to function adaptively or achieve success in the new environment, they may need to embrace new values and ideologies (Chen and Chen, 2010). Similarly, societal developments toward urbanization may shift cultural values, parental attitudes, and developmental pathways toward more independent social behavior and values (Chen et al., 2010), which may in turn influence the meaning and correlates of parenting strategies.

Since the early 1990s, China has experienced one of the most dramatic economic reforms, with transformational shifts taking place in major urban centers such as the capital, Beijing. There have been substantial increases in individual and family income, with average wages for urban residents rising almost 10-fold between 1995 and 2013, from 5,348 Yuan (USD 799) to 51,483 Yuan (USD 7,700) (National Bureau of Statistics of China, 2013). The rise of China to the world’s second largest economy represents a mercurial shift toward urbanization and commerce, resulting in the emergence of a large affluent class of families in urban cities. With these societal changes, there have been ripple effects in parents’ perceptions of social change, namely in their perceptions of transformations in work-related demands and needed competencies, and the experience of shifting values. These changes can ultimately affect their beliefs about how to rear their children in order to adapt to new ecological demands. Contemporary urban Chinese parents experiencing such social change have been described as shifting toward a more child-centered, independence-oriented form of parenting consistent with a general shift toward individualism (Chen et al., 2010).

While economic reform has provided unprecedented opportunities for people in the workforce, such as increased earnings, job and advancement opportunities, it nonetheless poses new challenges. Prior to economic reform, all workers and employers in China were matched to state-sponsored jobs by the government. Even though wages were state controlled and work mobility was limited, lifetime employment was guaranteed. As individuals enter the private sector for work, there is increased competition, greater uncertainty, and higher work-related risk. Given these shifts in the labor market and economy, parents may enhance their children’s chances for success by instilling traits historically valued in individualistic societies – such as assertiveness, confidence, and self-reliance (Chen and Chen, 2010). Along with economic growth came the import of technology, such as cell phones, computer, and the Internet. China surpassed the United States to become the world’s largest Internet user in 2008, with more than 70% of urban residents using the internet in 2013 (China Internet Network Information Center, 2014). Despite significant government censorship, the Internet has ushered in global cultural influence and communication in which foreign ideas and information are largely accessible to formerly closed mainland China (Hong, 1998). Indeed, compared to rural Chinese parents, urban Chinese mothers and fathers report increasing experience and need to interface with technology, and more opportunities and challenges associated with work-relater advancement (Chen et al., 2010). However, previous studies have not examined how parental perceptions these social changes are associated with parenting views and practices – an important predictor of how social change experiences are transmitted across generations.

These various global cultural changes and communication forces may shape value systems on both an individual and family level, which in turn shape socialization goals and parenting practices. Indeed, evidence suggests that cultural values and parenting are changing rapidly in mainland China with parents in urban settings exerting less control and granting greater autonomy to their children compared to parents in rural China owing to conditions of social change (Chen et al., 2010), with some variation in values explained by birth after the institution of the one-child policy (Zhan, 2004). Compared to mainland China, shifts in other parts of the Chinese diaspora have been longer-term and less dramatic. Hong Kong was a British colony from 1898 to 1997 and is now a Special Administrative Region (SAR) of the People’s Republic of China (PRC). Hong Kong experienced its largest economic growth between 1961 and 1997 having a 180-fold increase in its gross domestic product during that period of time (Yeung, 2008). While it remains a major center of trade and finance, the society and economy at large have experienced stable growth (Yau and Smetana, 2003). At the same time, Hong Kong is still strongly influenced by traditional Confucian culture and parenting styles and behaviors may not be as impacted by social and economic developments. Research that contrasts parenting in mainland China and Chinese societies that have not undergone changes in family policy or recent rapid economic growth suggest that contemporary mainland Chinese parents exert less parental authority and control than their Hong Kong and Taiwan counterparts (Berndt et al., 1993; Lai et al., 2000).

Following the U.S. Immigration and Nationality Act of 1965, an influx of Chinese workers and families began immigrating to the United States with 384,000 Chinese immigrants settling by 1980 predominantly from Hong Kong and Taiwan. Immigration from mainland China increased in the 1990’s following the easing of migration restrictions in the P.R.C, and by 2013, there were more than 2 million foreign-born Chinese Americans (Hooper and Batalova, 2015). While the United States has not experienced the same rapid urbanization and economic shift that mainland China has, immigration may have similar impacts on perceptions of social change and values at the individual level. It is plausible that the individual changes that we observe among sedentary individuals in a changing society are parallel to experiences within cohorts immigrating from a less economically developed context (such as mainland China prior to its rapid urbanization and globalization) to a more economically developed and Western cultural environments. Indeed, Chinese–American immigrant families may adapt to host cultural parenting norms, with parents who are more assimilated to the American culture tending to adopt more authoritative parenting styles and allowing their child to explore more freely (e.g., Park et al., 2010).

Yet, on the other hand, many overseas Chinese in North America have been described as adhering to a heritage culture that is “frozen at the time when they emigrated from their country of origin” (Giguere et al., 2010). Furthermore, dissonant acculturation, whereby immigrant parents acculturate more slowly than their offspring to host cultural norms, has been related to increased family conflict and escalating reliance on parental control (Lau, 2010; Nguyen et al., 2017). In fact, recent studies have found that compared to both Chinese American and European American parents, mainland Chinese parents are now less likely to display negative affect when exerting control with their young children (Camras et al., 2008). Parents in mainland China who have undergone recent rapid social change may respond with more autonomy supportive parenting to shifts toward individualistic ecological conditions (Way et al., 2013), whereas mothers in Los Angeles and Hong Kong may endorse higher levels of parental control and lower levels of autonomy support.

Given that cultural environments are not static, even within a specific cultural context, it must be treated dynamically in developmental research. Previous examinations of perceived social change have largely tapped into perceptions of economic reform and the “open door” policy among mainland Chinese families by measuring parents’ sense of how their employment related risks and opportunities appear to have changed as well as their sense of changing societal values and ideologies (e.g., Chen et al., 2010). Additional studies have examined parenting within the backdrop of rapid social change by asking mothers about their orientation to or away from traditional Chinese cultural values and not of perceptions of *change* in values (e.g., Xu et al., 2005). Currently missing is examination of how parents’ perceptions of their own change toward new values and ideologies may distinctly impact parenting in the contexts of both rapidly evolving urbanization and economic development or immigration to a Western developed nation. We examined children and their mothers who all share ethnic Chinese cultural heritage and urban residence, but who reside in three different societal contexts and are faced with different immediate socioeconomic contingencies. The study had three main aims. First, we contrasted levels of Chinese mothers’ reported use of parental control (psychological control and autonomy support), perceived social change in globalized values and knowledge, and child reported family climate (perceived child acceptance and family conflict) across the three contexts: the United States (Los Angeles), Hong Kong, and China (Beijing). We hypothesized that in contrast to Hong Kong mothers in a relatively stable Chinese society, urban Chinese mothers in Beijing and urban Chinese mothers in Los Angeles would report lower levels of parental control, higher levels of autonomy support, and higher levels of new values and knowledge. In terms of children’s perceptions of family climate, we predicted that bicultural children in immigrant families in Los Angeles would report greater intergenerational conflict and lower maternal acceptance than Chinese children in Hong Kong and Beijing.

Our second aim was to locate group differences in parental control within different ecological contexts facing families, such as parents’ perceptions of changes in values and knowledge as a result of broader social and economic developments. Specifically, we examined whether new values and knowledge would mediate differences in mother’s use of psychological control and autonomy support between Beijing, Hong Kong, and Los Angeles families. We predicted that parents’ endorsement of new values and knowledge would be associated with lower reports of psychological control and higher levels of autonomy support across groups, which may in part explain differences in application of parental control among urban Chinese families in different social contexts.

Our third and final aim was to examine potential variability and similarity in the associations between parental control and family relations among Chinese families in the three social contexts. We examined how psychological control and autonomy support related to children’s perceptions of family climate and tested whether these relationships differed across the three societal contexts. As described earlier, the cross-cultural parenting literature suggests a robust link between autonomy support and child and family functioning. In contrast, the results are more mixed concerning the correlates of parental psychological control, with only some studies showing attenuated risk in East Asian cultural contexts that prioritize family hierarchy and interdependence. Yet based on theories about the cultural variation in the meanings of parental control, the contexts of rapid social change in Beijing and bicultural adaptation in Los Angeles may result in normative shifts toward child autonomy that increase the risks to family climate associated with maternal psychological control compared to Hong Kong, where traditions in parental authority may be more stable.

## Materials and Methods

### Participants

Participants were 423 4th and 5th grade children and their mothers (142 dyads from Beijing, 150 dyads from Hong Kong, and 131 dyads from Los Angeles). For the Beijing (BJ) sample, child age ranged from 8 to 12 years (*M* = 9.88, *SD* = 0.79) and maternal age ranged from 32 to 65 years (*M* = 39.27, *SD* = 6.51). For the Hong Kong (HK) sample, child age ranged from 8 to 11 years (*M* = 9.65, *SD* = 0.69) and maternal age ranged from 29 to 63 years (*M* = 41.42, *SD* = 6.65). In the Los Angeles (LA) sample, child age ranged from 7 to 12 years (*M* = 9.61; *SD* = 0.98) and maternal age ranged from 32 to 59 years (*M* = 43.02; *SD* = 4.78). Approximately half of the child participants were female (49.66% in BJ, 50.34% in HK, and 56.25% in LA). Four mothers in our sample indicated maternal age above 60 years, approximately two standard deviations from mean maternal age for the sample. Due to concerns that outliers on maternal age may indicate that the respondent was the child’s grandmother or a mother from a different generational cohort, we elected to remove these four observations (1 from BJ and 3 from HK) for a total of 419 observations included in present analyses.

All participants were of Chinese-heritage and the majority of child participants were born in their area of residence (95.7% in BJ, 95.2% in HK, and 80.6% in LA). In terms of the mothers, 85.7% of mothers in BJ were both in their area of residence, compared to 77.5% in HK and 11.9% in LA. Among LA mothers who reported their birthplace, 40.5% were born in Mainland China, 10.3% in HK, 23.0% in Taiwan, 14.3% in other Asian countries, and 11.9% in the United States. For children in LA, 19.4% were first-generation Chinese Americans and 80.6% were at least second-generation Chinese American. A little less than half of LA families lived in historical ethnic enclaves with high Chinese ethnic family density (40.9%) and the remaining LA families hailed from other diverse neighborhoods around greater Los Angeles. In terms of maternal employment, 60.1% of BJ mothers worked at least 30 hours a week, versus 50% of HK mothers and 59.8% of LA mothers. In terms of maternal education, 78.8% of BJ mothers, compared to 31.6% of HK mothers and 78.1% of LA mothers received at least a 4-year college degree. In terms of family income, the median household income is between 37,200 and 60,999 RMB in BJ, 108,000 and 149,999 HKD in HK, and 75,000 and 124,000 USD in LA.

According to the 2015 United States Census, Los Angeles is a high-density city (8,474 people per square mile) and is ethnically diverse (28% European American, 9% African American, 11% Asian American, and 49% Hispanic American). According to the 2012 United States Census Bureau, 30% of the population over the age of 25 having at least a 4-year college degree and a median annual family gross income of $57,271. Similarly, Beijing is a high-density city (12,800 people per square mile) with 36% of the population over the age of 15 having at least a 4-year college degree and average annual wage of employed persons in urban private unit of 34,235 RMB (Beijing Municipal Statistical Bureau, 2011). Hong Kong is densely populated (16,599 people per square mile) with 27% of the population over the age of 15 having at least a 4-year college degree and a median annual family gross income of 246,000 HKD. Participants were recruited through one local elementary school in BJ, one local elementary school in HK, and one local elementary school and two local weekend Chinese-heritage language schools in LA with the permission and administrative help of school officials. The selected elementary schools were considered average or typical schools; no schools were reputed to be particularly high- or low-achieving.

### Procedure

Students and parents were informed that the study was an international collaboration studying parenting among Chinese-descent families and child adjustment. Children were given study packets, which included consent and questionnaires to take home to discuss with their parents and complete the questionnaires if they decided to participate in the study. BJ children and parents were provided questionnaires in simplified Chinese and HK children and parents were provided questionnaires in traditional Chinese. The assessment battery included measures with previously established reliability with Chinese immigrant as well as national Chinese parents in HK and BJ. All instruments underwent translation, back-translation, and consensus reconciliation for conceptual equivalence. LA children completed their surveys in English while mothers completed the surveys in their preferred written language (English, simplified Chinese, or traditional Chinese). All children and their mothers completed questionnaires independently.

### Measures

#### Psychological Control

Mothers reported their use of psychological control by responding to the 18-item Psychological Control scale (Wang et al., 2007). The scale included 10 statements that measured *guilt induction* (e.g., “I tell my child that he/she should feel guilty when he/she does not meet my expectations.”), 5 statements that measured *love withdrawal* (e.g., “I act cold and unfriendly if my child does something I do not like.”), and 3 statements that measured *authority assertion* (e.g., “I tell my child that what I want him/her to do is the best for him/her and he/she should not question it.”). Mothers indicated level of agreement with each statement on a 5-point Likert scale (0 = *not at all true*; 4 = *very true*). The Chinese translation of the measure has been used among parents in Beijing with good internal reliability (Wang et al., 2007; α = 0.92), an abbreviated 12-item of the measure has also been used in a sample of Hong Kong parents (Fung and Lau, 2012; α = 0.75). The overall scale yielded good internal consistency for the current sample with Cronbach alphas being 0.92 in BJ, 0.94 in HK, and 0.91 in LA.

#### Autonomy Support

Mothers’ use of autonomy supportive parenting practices was measured using the 12-item Autonomy Support scale (Wang et al., 2007). Six items tapped into *opinion exchange* (e.g., “I encourage my child to give ideas and opinions when it comes to decisions about him/her.”) and six items tapped into *choice making* (e.g., “I allow my child to make choices whenever possible.”). Mothers responded with her level of agreement for each statement on a 5-point Likert scale (0 = *not at all true*; 4 = *very true*). The Chinese translation of the measure has been used among parents in Beijing with good internal consistency (Wang et al., 2007; α = 0.90). The overall scale yielded good internal consistency with Cronbach alphas being 0.89, 0.93, and 0.88 for BJ, HK, and LA, respectively.

#### Perceived Social Change

Mothers reported their perception of social changes within the last 5 years by responding to the 22-item Perceived Social Change scale (Chen et al., 2010). The original scale assesses individual perceptions of socio-economic context on five factors: work-related risk and adversity, work-related opportunities, self-improvement in work, experiences of high-technology, and new values and knowledge. Given the study’s focus on parenting behaviors, we elected to focus on the subscale, *New values and knowledge*, that highlights individual experiences of new values and ideologies as a function of social change (e.g., “The knowledge and experience of my parents offer fewer clues for my life today.”, “My views and values are more influenced by modern Western societies, like America.”, “Old values and habits from the past offer little guidance for my situation today.”). Mothers rated each item on a 5-point Likert scale (0 = *not at all true*; 4 = *very true*). The scaled yield good internal reliability (α = 0.64) in previous studies among parents in Beijing (Chen et al., 2010). Internal reliabilities for the subscale was 0.77, 0.84, and 0.80 for BJ, HK, and LA, respectively.

#### Child Acceptance

Children’s perceived maternal acceptance was assessed by the 10-item acceptance–rejection subscale of the Children’s Report of Parent Behavior Inventory (Schaefer, 1965; Rohner, 1984). These items were modified to ask about children’s perceived affection and responsiveness from their mothers (e.g., “She smiles at me very often.”). Children in LA indicated their level of agreement with each statement on a 3-point Likert scale (0 = *not like her*; 2 = *like her*) whereas children in BJ and HK responded using a 5-point Likert scale (0 = *totally disagree*; 4 = *totally agree*). To address the rating scale discrepancy between the three cities, weighted means were used in all analyses. The subscale has demonstrated good internal consistency with alphas of 0.88 for Chinese Americans (Wu and Chao, 2005) and 0.92 for Chinese adolescents in Beijing (Quach et al., 2015). For the current sample, the scale has good internal reliability with Cronbach alphas being 0.86 in BJ, 0.94 in HK, and 0.88 in LA.

#### Family Conflict

The occurrence of intergenerational conflict between the child and the mother was measured by the Asian American Family Conflict Scale-Likelihood scale (Lee et al., 2000). Children responded to 10 statements concerning the likelihood and severity of disagreements concerning parental control versus child autonomy (e.g., “Your mother tells you what to do with your life, but you want to make your own decisions.”) and the nature of the parent–child relationship (e.g., “Your mother always compares you to others, but you want them to accept you for being yourself.”). The scale originally referred to parents in general and was modified in the current study to describe disagreements occurring between the mother and the child. Children used a 5-point Likert scale (0 = *almost never*; 4 = *almost always*) to rate the likelihood of these situations occurring in their family. While children’s ratings may vary for a variety of different reasons, the scale has been used as a reliable measure of the frequency of parent–child conflict among Beijing students (Miller and Lee, 2009; α = 0.78) and Chinese immigrant youths in the United States (Ma and Yeh, 2005; α = 0.83). Cronbach alphas of the child report of family conflict were 0.79 in BJ, 0.86 in HK, and 0.91 in LA.

## Results

### Aim 1 Preliminary Analyses

Descriptive statistics for the main study variables are listed in **Table 1**. One-way ANOVAs revealed significant group mean differences on all main variables of interest. In particular, Chinese American mothers in Los Angeles (LA) reported significantly higher perceived New Values and Knowledge than mothers in Hong Kong (HK) (*t* = -3.47, *p* < 0.001) and Beijing (*t* = -3.58, *p* < 0.001).

**Table 1:** Means and standard deviations of social change, parental control, and family relations by city.

	Beijing		Hong Kong		Los Angeles		
				
	*M*	*SD*	*M*	*SD*	*M*	*SD*	*F*
New values and knowledge	8.22^a^	3.30	8.24^a^	3.60	9.93^c^	4.38	8.87^∗∗^
Psychological control	1.42^a^	0.59	1.69^b^	0.67	1.23^c^	0.65	18.13^∗∗∗^
Autonomy support	2.65^a^	0.57	2.42^b^	0.63	2.57^ab^	0.59	5.70^∗∗^
Family conflict	1.71^a^	0.80	1.84^a^	0.91	1.27^c^	1.01	13.11^∗∗∗^
Perceived acceptance^∧^	2.62^a^	0.78	2.74^ab^	0.96	2.94^b^	0.89	4.10^∗^


*^∧^Means on the acceptance measure were weighted in Los Angeles to be able to compare means across cities. Means with different superscripts denote significantly different means. ^ab^Mean is not significantly different from means for the other two cities. ^∗^*p* < 0.05, ^∗∗^*p* < 0.01, ^∗∗∗^*p* < 0.001.*

In terms of parenting, HK mothers reported significantly higher use of Psychological Control than BJ mothers (*t* = -3.55, *p* < 0.001) who reported significantly higher use of Psychological Control than LA mothers (*t* = 2.55, *p* < 0.01). BJ mothers reported significantly greater use of Autonomy Support than HK mothers (*t* = 3.29, *p* < 0.001). Report of autonomy support among LA mothers did not significantly differ from the report of Autonomy Support in BJ (*t* = -1.12, *p* > 0.05) or HK (*t* = -2.31, *p* < 0.10).

Regarding family climate, children in LA also reported significantly lower Family Conflict and higher Child Acceptance compared to their BJ (*t* = 3.78, *p* < 0.001 and *t* = -3.03, *p* < 0.01, respectively). However, compared with their HK counterparts, LA children reported significantly lower Family Conflict (*t* = 4.73, *p* < 0.001), but not significantly different perceptions of Child Acceptance (*t* = -1.67, *p* > 0.05).

**Table 2** lists the table of correlations by each of the three cities for all main variables of interest. New Values and Knowledge was positively and significantly correlated with Autonomy Support in HK, but not in BJ or LA. New Values and Knowledge was positively associated with Psychological Control in HK and LA, but not in BJ. The relationship between mother’s report of Psychological Control and Autonomy Support was negative in BJ, positive in HK, and non-significant in LA. Psychological Control was positively correlated with Family Conflict in BJ and LA and negatively associated with Child Acceptance in LA only. Autonomy Support was associated with decreased Family Conflict in LA. Autonomy Support was significantly associated with increased Child Acceptance in HK and LA and marginally associated in BJ. Finally, the relationship between Child Acceptance and Family Conflict was positive for HK, negative for LA, and non-significant for BJ families.

**Table 2:** Pairwise correlations for variables in Beijing sample (A), Hong Kong sample (B), and Los Angeles sample (C).

Variable	1	2	3	4	5
**(A) Beijing sample**				
(1) New values and knowledge	–				
(2) Psychological control (mother report)	-0.03	–			
(3) Autonomy support (mother report)	0.11	-0.30***	–		
(4) Family conflict (child report)	0.03	0.27**	0.05	–	
(5) Perceived acceptance (child report)	0.14	-0.10	0.16^†^	0.01	–
**(B) Hong Kong sample**
(1) New values and knowledge	–				
(2) Psychological control (mother report)	0.48***	–			
(3) Autonomy support (mother report)	0.17*	0.27**	–		
(4) Family conflict (child report)	-0.03	0.08	0.10	–	
(5) Perceived acceptance (child report)	0.13	0.02	0.20*	0.23**	–
**(C) Los Angeles sample**
(1) New values and knowledge	–				
(2) Psychological control (mother report)	0.32***	–			
(3) Autonomy support (mother report)	0.04	-0.02	–		
(4) Family conflict (child report)	0.19^†^	0.40***	-0.20*	–	
(5) Perceived acceptance (child report)	-0.11	-0.19*	0.27**	-0.31**	–


*^†^*p* < 0.10, ^∗^*p* < 0.05, ^∗∗^*p* < 0.01, ^∗∗∗^*p* < 0.001.*

### Aim 2 Analyses: Mediation Analysis

A series of hierarchical regression analyses were used to test the hypothesis that group differences in Psychological Control and Autonomy Support would be mediated by perceived Social Change (Baron and Kenny, 1986). HK was used as the reference group, with two dummy variables created for the BJ and LA samples. Results of the analyses are shown in **Table 3**. After controlling for child’s age, child’s gender, and maternal education, the dummy variables for BJ and LA were significantly associated with Psychological Control (β = -0.14, *p* < 0.05 and β = -0.21, *p* < 0.01, respectively) and Autonomy Support (β = 0.24, *p* < 0.001 and β = 0.15, *p* < 0.05, respectively), suggesting that BJ and LA mothers reported using more Psychological Control and Autonomy Support strategies compared to HK mothers. In the second step of the model, BJ and LA were simultaneously regressed on parental control along with New Values and Knowledge and maternal education. Separate models were run looking at Psychological Control and Autonomy Support. Sobel’s (1982) test was conducted to demonstrate that when controlling for New Values and Knowledge, group differences in Psychological Control and Autonomy Support are attenuated.

**Table 3:** Summary of two hierarchical regression analyses evaluating perceived social change as a potential mediator of group differences in psychological control and autonomy support.

	*B*	*SE B*	β	*R*^2^
**DV: Psychological control**				
Step 1
Child sex	0.002	0.07	0.002
Child age	0.05	0.04	0.06
Mother’s education	-0.02	0.01	-0.16**
Beijing^a^	-0.20	0.09	-0.14*
Los Angeles^a^	-0.31	0.10	-0.21**	0.10***
Step 2
Mother’s education	-0.03	0.01	-0.22***
Beijing^a^	-0.16	0.08	-0.12*
Los Angeles^a^	-0.38	0.09	-0.27***
New values and knowledge	0.05	0.01	0.29***	0.20***
**DV: Autonomy support**				
Step 1
Child sex	0.06	0.07	0.05
Child age	0.0002	0.04	0.0003	
Mother’s education	-0.004	0.01	-0.03
Beijing^a^	0.31	0.09	0.24***
Los Angeles^a^	0.20	0.09	0.15*	0.04*
Step 2
Mother’s education	-0.003	0.01	-0.02
Beijing^a^	0.26	0.08	0.20**
Los Angeles^a^	0.11	0.09	0.08
New values and knowledge	0.02	0.01	0.10^†^	0.04**


*Reference group: ^a^Hong Kong. ^†^*p* < 0.10, ^∗^*p* < 0.05, ^∗∗^*p* < 0.01, ^∗∗∗^*p* < 0.001.*

**FIGURE 1:**
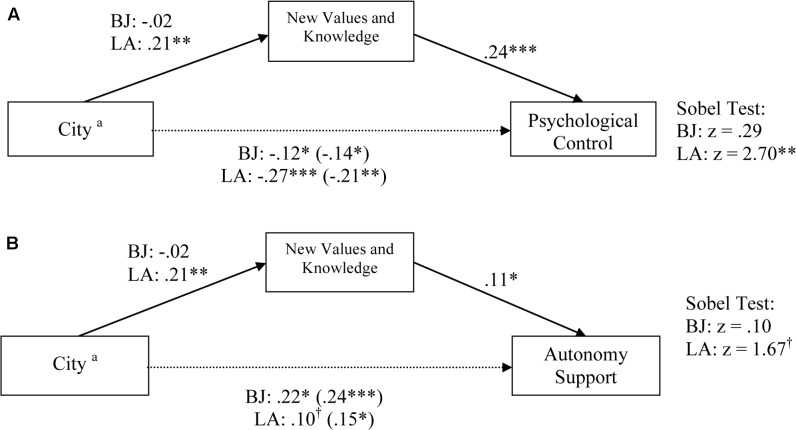
**(A)** New values and knowledge as a suppressor of the group differences in psychological control in Los Angeles. **(B)** New values and knowledge as a partial mediator of the group differences in autonomy support in Los Angeles. Reference group: ^a^Hong Kong. Child sex, child age, mother age, and maternal education level were entered into the model as covariates. ^†^*p* < 0.06, ^∗^*p* < 0.05, ^∗∗^*p* < 0.01, ^∗∗∗^*p* < 0.001.

For Autonomy Support, as shown in **Figure 1B**, the regression coefficient for the LA dummy variable (β = 0.10, *p* < 0.10) was attenuated and marginal when New Values and Knowledge was simultaneously regressed on Autonomy Support. The mediated effect on Autonomy Support was marginal for LA (*z* = 1.67, *p* < 0.10). However, as shown in **Figure 1A**, the mediation analyses revealed evidence of statistical suppression in the model interrogating Psychological Control for LA. The regression coefficient for the LA dummy variable increased (from β = -0.21, *p* < 0.01 to β = -0.27, *p* < 0.001) after the inclusion of New Values and Knowledge as a predictor of Psychological Control, suggesting suppression effects. The suppression effects of New Values and Knowledge on Autonomy Support was significant for LA (*z* = 2.70, *p* < 0.01).

### Aim 3 Analyses: Multiple-Group Path Analysis

**FIGURE 2:**
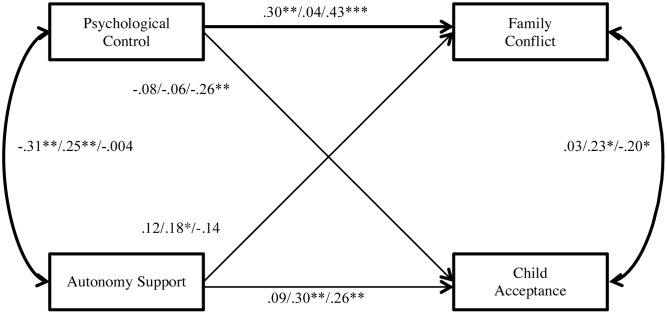
Multiple-group path analysis of parenting and family process variables. Standardized coefficients for each city is depicting following the format Beijing/Hong Kong/Los Angeles. Paths that are variant across groups are depicted in bold. Child sex, child age, mother age, and maternal education level were entered into the model as covariates. ^†^*p* < 0.06, ^∗^*p* < 0.05, ^∗∗^*p* < 0.01, ^∗∗∗^*p* < 0.001.

A multiple-group path analysis was then employed to test the hypothesized relationships between Psychological Control, Autonomy Support, Family Conflict, and Child Acceptance among the variables of interest as well as to test if they were equivalent across cities (**Figure 2**). The statistical package Mplus 7.0 was used for the analysis (Muthén and Muthén, 1998–2012). Structural equation modeling (SEM) was used with maximum likelihood estimation to create a multiple-group model (Brown, 2012). Full information maximum likelihood was utilized in order to include all observations in the estimation of parameters, including observations missing data on one or more variables of interest. Maternal report of Psychology Control and Autonomy Support and child report of Family Conflict and Child Acceptance were tested for their association with one another. This model displayed good fit with RMSEA = 0.05, CFI = 0.88, TLI = 0.83, and SRMR = 0.05. A chi-square difference test was utilized to determine whether there was a significant difference between the constrained and unconstrained model, such that a significant change in chi-square value between the two models indicated that the parameter estimate differed between groups. Results of the chi-square difference test (χ^2^(10) = 35.87; *p* < 0.001) indicated that it is most appropriate to examine data within a multiple-group model rather than a single group model.

The model indicated that maternal Psychological Control was positively significantly associated with child perceived Family Conflict for BJ (β_BJ_ = 0.30, *p* < 0.01) and LA (β_LA_ = 0.43, *p* < 0.001), but not for HK. A chi-square difference test revealed that the path differed significantly across the three groups (χ^2^(2) = 8.85; *p* < 0.05). Maternal Psychological Control was also significantly negatively correlated with Child Acceptance in LA (β_LA_ = -0.26, *p* < 0.01), and not in BJ or HK. In terms of Autonomy Support, maternal Autonomy Support was significantly associated with perceived Child Acceptance for HK (β_HK_ = 0.30, *p* < 0.01) and LA (β_LA_ = 0.26, *p* < 0.01) families, but not for BJ families. Autonomy Support was significantly associated with Family Conflict in HK only (β_HK_ = 0.18, *p* < 0.05). Mother report of Autonomy Support and Psychological Control were significantly negatively correlated in BJ (β_BJ_ = -0.31, *p* < 0.01), significantly positively correlated in HK (β_HK_ = 0.25, *p* < 0.01), and not significantly correlated amongst LA families (β_LA_ = 0.004, *p* > 0.05; χ^2^(2) = 16.21; *p* < 0.001). Finally, child report of Family Conflict and Child Acceptance were significantly negatively correlated in LA (β_LA_ = -0.20, *p* < 0.05), positively correlated in HK (β_HK_ = 0.23, *p* > 0.05), and not significantly correlated in BJ; the path differed significantly across groups (χ^2^(2) = 7.73; *p* < 0.05).

## Discussion

The first study goal was to directly compare levels of parental control, perceived social change and family climate between families of Chinese descent residing in three urban cities but with different immediate social and economic contingencies: the United States (Los Angeles), Hong Kong, and mainland China (Beijing). Consistent with our hypothesis, Hong Kong (HK) mothers demonstrated greatest use of psychological control compared with mothers in Beijing (BJ) and Los Angeles (LA) and lower use of autonomy support than mothers in BJ. Despite the high level of economic development in HK, meta-analytic evidence suggests that HK residents are significantly less individualistic compared with European–Americans (Oyserman et al., 2002). Confucian traditions in child socialization appear to be entrenched and stable in HK where families endorsed the highest levels of restrictive control and lowest levels of autonomy support. Our results are consistent with recent comparative studies that found HK mothers to be more authoritarian and controlling than BJ mothers (e.g., Lai et al., 2000), suggesting that psychological control may continue to be viewed as an acceptable and effective form of socialization in HK. On the other hand, LA mothers endorsed the lowest levels of psychological control. This may suggest acculturation related movement toward Western norms and expectations and less adherence to traditional Chinese child-rearing styles of invoking parental authority. Our finding suggests that Chinese immigrant parents may adopt United States parenting values and norms, more so than experiencing “heritage cultural freezing” (Giguere et al., 2010) or escalating control in the face of intergenerational acculturation dissonance (Nguyen et al., 2017). BJ mothers also endorsed greater use of autonomy support than HK mothers. This is consistent with our hypothesis and recent studies documenting a shift in cultural values and ideals in mainland urban China toward valuing children’s autonomous and self-directive behaviors (Chen et al., 2010) and exerting less parental authority and restrictive control (Lai et al., 2000).

Contrary to our prediction, Chinese immigrant mothers in LA reported the highest levels of perceived social change (as measured by their endorsement of values, knowledge, and lifestyle reflecting new norms instead of traditional ideology and habits). More than half of the Chinese immigrant mothers in LA in our sample are foreign-born, thus it is reasonable to expect that levels of perceived social change is high in LA as immigrant mothers may have experienced greater exposure to new ideas and values as a function of their migration to the United States. The overall pattern of high perceived social change and reduced reliance on psychological control and more autonomy support may reflect Chinese immigrant mothers’ acculturation toward the United States cultural context. To the extent that LA mothers adopt new values and ideals, they acculturate and take on the behaviors, values, and attitudes of the host culture including the domain of parenting (Berry et al., 1986). Interestingly, qualitative research with Somali, Sudanese, and Ethiopian families who had migrated to Australia illustrate patterns in which parents practice increasingly restrictive parenting and obstruct youths’ autonomous decision making (Renzaho et al., 2011), which may suggest that the combination of social change and migration (and not just migration alone), is a key facilitator of the association from social change to shifts in parenting behaviors.

In terms of family climate, we predicted that Chinese American children would more frequently perceive conflicts with parents compared to children in HK or BJ possibly due to different rates at which children and parents acculturate, thus resulting in increased family conflict (e.g., Portes and Rumbaut, 2006). However, contrary to our prediction, children in LA reported the lowest levels of family conflict and higher levels of perceived acceptance compared with children in BJ, with no significant difference between LA and the other cities. Most work that examines acculturation dissonance and family conflict focuses on adolescents (e.g., Harker, 2001). Our study, on the other hand, involves mothers who seem to be acclimating to the United States culture with elementary school aged children. It is possible that the acculturation gap between mothers and children in our sample may be narrower, which may explain why we found lower conflict and greater perceived acceptance. Furthermore, we sampled from among Chinese American families who was either living in relatively enclave neighborhoods, or who had their children enrolled in heritage language classes. As such, these families may represent families that are navigating a balance between acculturation and maintaining enculturation to family interdependence values that dampen the risks of conflict related to acculturation dissonance. Indeed, some research on Chinese immigrant families suggests that the risks of family discord are greatest when there are gaps in levels of enculturation rather than acculturation between parents and children (Costigan and Dokis, 2006; Telzer, 2011).

The second study goal was to examine whether differences in parental control across the three cities may be due to differences in mothers’ perceived social change. Consistent with theoretical predictions, perceived social change was significantly associated with higher levels of mother’s report of autonomy support in all three cities, but results of the mediation analyses suggest that perceived social change mediated group differences in maternal use of autonomy support among LA families only. To the extent that mothers in LA reported being more influenced by the introduction of new values and knowledge, they were more likely to encourage their children to express their individuality or make independent choices. As such, group differences in the use of autonomy support could in part be explained by perceived social change. However, somewhat surprisingly, in examining group differences in psychological control across groups we observed suppression effects (MacKinnon et al., 2007). Rather than showing mediation, differences in mother’s use of psychological control within LA were even greater when taking into account perceived social change. Certainly, LA mothers reported higher levels of perceived social change in the form of new values and knowledge. Because perceived social change was positively associated with psychological control, LA mothers reporting lower levels of psychological control thus reflect somewhat of a paradox. This may suggest that compared to autonomy support, the use and meaning of psychological control may be more nuanced and may suggest the role of other variables (e.g., maternal education) in understanding group differences among Chinese American mothers.

In terms of the relationship between perceived social change and mother’s parenting practices, our findings suggested that the more mothers reported being influenced by social change, the more they felt the need to both exert control and encourage independence in their children. Two theoretical models have recently been put forth regarding the relationship between social change and cultural values. Whereas Greenfield (2009) proposes that economic development and urbanization lead to declines in interdependence and rises in independence values, Kagitcibasi (2005, 2013) argues that interdependence can be maintained despite new emphasis on independence, especially in cultures that are traditionally interdependent. Specifically, Kagitcibasi makes a distinction between autonomy (as opposed to heteronomy) and separation (as opposed to relatedness), and asserts that intergenerational psychological relatedness can remain even when personal or behavioral autonomy increases. Overall, our data appears to be aligned with Kagitcibasi’s notion that social change may result in both the promotion of independence values (self-efficacy via autonomy support) and retention of traditional values (relatedness via psychological control). Indeed, studies of other cultures exhibit similar patterns of maintaining interdependent values even as autonomy increases. For instance, Park et al. (2015) found that while rapid social change in South Korean led to the dissolving of group differences between urban and rural Koreans’ individualistic values, Korean immigrants dually displayed collectivistic values in the home but individualistic values at school.

The third goal was to contrast the associations between parental control and family climate among families from BJ, HK, and LA. Our data found both similarities and differences in how psychological control and autonomy support relate to family process across the three cultural contexts. Firstly, the association between psychological control and family conflict differed significantly between the three cities with psychological control associated with higher levels of family conflict in BJ and LA, but the relationship was not significant for HK. Similar to findings observed in studies of Western families (e.g., Garber and Flynn, 2001), psychological control was associated with children’s perceptions of lower maternal acceptance among LA families. Overall, this suggests that Chinese immigrant parents may not only adopt United States parenting values and norms, but that the implications of parental control as perceived by the child may be more similar to that observed in Western families.

Our null relationship between psychological control and family processes in HK is somewhat different from previous studies that found parental psychological control to predict lower parent–child relational quality among adolescents (Shek, 2006). The difference may in part be due to the fact that Shek (2006) examined the relationship among adolescents and used measures that capture constructs of trust and communication rather than warmth and conflict. This pattern of Chinese socialization often changes as a function of the child’s age with Chinese parents tending be more lenient and permissive toward younger children and instead impose more restrictive discipline upon older children (Ho, 1986). Furthermore, psychological control strategies are often conducted in the context of, and with the tacit goal of, inducing emotional closeness between the parent and child within Chinese-heritage families (Wu, 1981). Thus, younger children may be more likely to perceive criticism and correction as reflective of parental concern or involvement rather than acts of hostility or rejection (Chao, 1995). As such, in the future, researchers might investigate how these patterns of associations may differ based on age. Autonomy support was significantly associated with family conflict only in HK and was associated with higher levels of perceived child acceptance in HK and LA. Finally, the relationship between psychological control and autonomy support differed significantly by societal context. There was a null relationship between psychological control and autonomy support in LA, whereas the relationship was significantly positive in HK and significantly negative in BJ. Some scholars have argued that, rather than being on the opposite end of a single dimension, psychological control and autonomy support should be viewed as two independent constructs with distinct pathways to child’s development (Barber and Harmon, 2002; Silk et al., 2003; Wang et al., 2007; Kim et al., 2017). Our study suggests that the relationships between these two forms of parental control are nuanced, and may depend on the broader cultural and societal context. Our empirical data supported the aforementioned notion that parent’s use of one control strategy (psychological control) was independent of and distinct from the other (autonomy support), but only in LA.

We observed very different patterns of association between psychological control and autonomy support in HK and BJ. In HK, the more mothers used psychological control strategies of shaming or love withdrawal, the more they also encouraged their child to make choices or express their opinions. On the other hand, the more BJ mothers used guilt induction and love withdrawal, the less they supported their child’s autonomy or individuality. HK is a cosmopolitan city that has enjoyed modern developments over the past decades (Ralston et al., 2006). Perhaps due to the intermixed influences of both the Chinese culture and British colonialism, HK retains traditional Chinese Confucian ideas of filial piety, familial hierarchy and affiliative competence while adopting modern ideas from the West. As such, we see mothers in HK embracing and balancing both aspects of parental control simultaneously. As HK mothers socialize their child to be cognizant of family order and social rules, they also encourage their child to develop assertive, independent and autonomous behaviors. On the other hand, our study data found psychological control to be antithetical to autonomy support among BJ mothers. As BJ mothers allow and support their child to make more independent choices, they are less likely to constrain their child’s self-expression by asserting parental authority. It is possible that BJ mothers feel that psychological control strategies are incompatible with new values and parental ideals of self-expression, exploration, and individuality that they try to instill in their child to help them succeed in the new and more competitive economy.

Overall, the present study revealed significant differences in the use of psychological control and autonomy support among BJ, HK, and LA mothers and that these differences may be in part rooted in perceived social change. Individual experiences of new values and ideologies as a function of social change was found to be associated with increased use of autonomy support as well as psychological control. This suggests that as families undergo economic and social change, there may be a shift to greater reliance of strategies that promote child’s autonomy and self-reliance, while at the same time retaining strategies that emphasize more traditional Chinese values of relatedness. Our study also revealed both similarities and differences in terms of the associations between parental control and family processes among Chinese families in different societal contexts. Whereas autonomy support was generally linked to favorable outcome of perceived child acceptance in HK and LA, psychological control was associated with more detrimental outcome of increased family conflict in BJ and LA. Overall, our findings revealed nuances regarding urbanization, immigration experiences and parental control strategies. It appears that as families undergo urbanization or social change either through change within their native country or via migration, it may shift the implications of traditional strategies that are intended to socialize the child toward interpersonal attunement and affiliative competence. Finally, our study demonstrated preliminary support that parental control may have direct implications on family climate outcomes.

There were several limitations in the study that must be acknowledged. First, because the study was cross-sectional and correlational, we cannot draw inferences about cause or directionality of effects. For example, parents may feel a greater need to exert restrictive control when there are existing tensions and conflict in the family. It is possible that children who feel accepted by their parents and experience positive parent–child relationships are more likely to elicit behaviors from the parents that encourage the child’s sense of autonomy. Second, although the group comparisons were selected to make specific theoretical contrasts, it is difficult to attribute differences directly to processes of social change or acculturation as selection effects may be at play. For instance, Chinese American parents may rely less on control and grant more autonomy than the other groups because they were predisposed to values of independence in ways that initially encouraged their decision to immigrate West. Third, our measures were limited to mother self-reports. The impact of father’s use of control strategies needs to be explored in future research, especially since recent studies found that Chinese paternal parenting predicting child outcomes above and beyond maternal parenting styles (Nelson and Carson, 2006). Furthermore, economic growth and social change may exert a greater impact on fathers more than mothers (Chen et al., 2010) as fathers are traditionally viewed as primary breadwinners. As such, fathers might be more impacted by social or economic change and the resultant shift in values and ideals. To improve the validity of our findings, future research should adopt multi-method and multi-discipline approach in understanding the nuances of social change, parenting practices, and family processes. Fourth, our studies found that perceived social change was related to maternal education attainment in BJ, though not in HK or LA. As such, some of the predicted differences between BJ and HK in particular were accounted for by differences in maternal education. In our attempts to unpack the unique contribution of perceived social change we included maternal education as a covariate in analyses and likely contributed to our inability to detect significant group differences between HK and BJ. Given that BJ is still undergoing rapid economic growth and social development, it is important to continue to examine possible interaction effects wherein families in Mainland China may be differentially impacted by the effects of social change. The implications of traditional control strategies may continue to shift as a function of parental education as well as broader social and economic development and change. Fifth, we operationalized perceived social change in the present study as mothers’ endorsement of having new values and ideologies different over the past 5 years. In addition to mothers who are experiencing changing societies, mothers who have moved or were bicultural may also rate higher on the perceived social change scale and unfortunately the present measurement does allow for unpacking the two. Also, the measure asked mothers to reflect on the past 5 years, as a way to measure more proximal perceptions of social change that has been occurring since the 1990s. As such, we can only conclude that perceptions of change ongoing within the past 5 years were associated with parental control. Finally, implications for child well-being should be extended beyond family conflict and perceived acceptance to examine implications for child adjustment in different socioeconomic contexts or in societies undergoing social change.

Despite these limitations, this is the first study to directly compare the relationships between parental control and family processes among families of Chinese descent who reside in different societal contexts. In particular, this study contributes to the current literature by moving beyond ethnic-group or cross-national comparisons to investigate the role of societal contexts in understanding differences in the reliance on parental control and its association with family process.

## Ethics Statement

The study was approved by the Institutional Review Board at University of California, Los Angeles.

## Author Contributions

JF and AL are the PIs of the study who oversaw the project and provided intellectual leadership. JK, QW, and CF oversaw recruitment and data collection in the United States, Hong Kong, and Benin, respectively. Finally, JK and JJ assisted with data analyses and manuscript preparation.

## Conflict of Interest Statement

The authors declare that the research was conducted in the absence of any commercial or financial relationships that could be construed as a potential conflict of interest.
